# Prevalence, incidence and predictive factors for hand eczema in young adults – a follow-up study

**DOI:** 10.1186/1471-5945-13-14

**Published:** 2013-10-29

**Authors:** Arne Johannisson, Ann Pontén, Åke Svensson

**Affiliations:** 1 Department of Health Sciences, Lund University, Box 157, 221 00 Lund, Sweden; 2 Department of Occupational and Environmental Dermatology, Lund University, Malmö, Sweden; 3 Department of Occupational and Environmental Dermatology, Malmö University Hospital, Malmö, Sweden; 4 Department of Dermatology, Lund University, Malmö, Sweden; 5 Department of Dermatology, Malmö University Hospital, Malmö, Sweden

**Keywords:** Hand eczema, Childhood eczema, Prevalence, Incidence, Cohort, Gender, Skin care, Hand-wash

## Abstract

**Background:**

Hand eczema is common in the general population and affects women twice as often as men. It is also the most frequent occupational skin disease. The economic consequences are considerable for society and for the affected individuals.

**Methods:**

To investigate the prevalence and incidence of hand eczema and to evaluate risk factors for development of hand eczema in young adults. Subjects and methods; This is a prospective follow-up study of 2,403 young adults, 16 – 19 years old in 1995 and aged 29 – 32 years, 13 years later, in 2008. They completed a postal questionnaire that included questions regarding one-year prevalence of hand eczema, childhood eczema, asthma, rhino-conjunctivitis and factors considered to affect hand eczema such as hand-washing, washing and cleaning, cooking, taking care of small children and usage of moisturisers. These factors were evaluated with the multinominal logistic regression analysis.

**Results:**

The one-year prevalence of hand eczema was 15.8% (females 20.3% and males 10.0%, p < 0.001). The incidence was 11.6 cases per 1000 person-years (females 14.3 and males 5.2, p < 0.001). Childhood eczema was the most important risk factor for hand eczema. The odds ratios were 13.17 when having hand eczema 1995 and 2008 compared to 5.17 in 2008 (p < 0.001). A high frequency of hand washing was important in predicting hand eczema only when having 1-year prevalence 2008, OR 1.02 (p = 0.038).

**Conclusions:**

After 13 years an increased 1-year prevalence of hand eczema was found. The significant risk factors for hand eczema changed over time from endogenous to exogenous factors.

## Background

Hand eczema is common in the general population. In a recent review of studies in the general population from mostly European countries, the 1-year prevalence rates ranged from 6.5% to 17.5% [1]. Hand eczema is 1.5 – 2 times more common in females compared with males [2,3]. Swedish estimates of 1-year prevalence of hand eczema in different age-groups have varied from 6.5% to 11.8% [4-6]. Among Swedish 20–29 year-olds, the 1-year prevalence of hand eczema was reported to range from 7.5% to 10.8% [3,4]. Furthermore, hand eczema is the most common occupational skin disease [7].

Occupation-related hand eczema has many negative consequences. The economic costs are considerable for affected individuals and for society [8,9]. Hand eczema has been shown to have an unfavourable long-term prognosis [10] and to impair quality of life [11]. These consequences could be reduced by identifying and preventing risk factors.

Several exogenous risk factors for hand eczema have been reported: occupational exposure, use of detergents and wet work at home [4,12-14]. The identification and evaluation of risk factors for the development and persistence of hand eczema are important especially among young adults. During this period of life, type of occupation, household work and childcare are factors that are important to study because they might be related to the development of hand eczema. Taken together, these circumstances justify follow-up studies in early adulthood.

The aim of the present study was to investigate the prevalence and cumulative incidence of hand eczema and to evaluate factors that can influence the development and recurrence of hand eczema in young adults.

## Methods

### Study group

**Figure 1:**
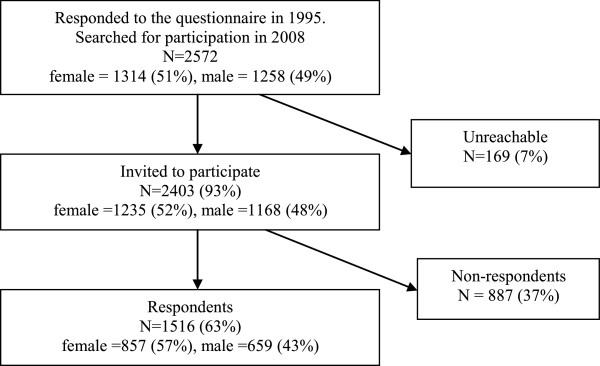
The flow-chart of the cohort.

This is the 13 year prospective follow-up study of a cohort of pupils in upper secondary school, 16–19 years old at the baseline assessment, and consequently they were 29–32 years old at follow-up. In 1995, 2,572 pupils in the four secondary schools in Växjö completed a self-administrated questionnaire regarding hand eczema, the response rate was 98.6%. Växjö is a town in southern Sweden with approximately 70,000 inhabitants [15,16]. In 1995, 74% of 16 – 19 years-olds attended secondary school in the study area, which was consistent with the overall attendance rate in Sweden. The 13-year follow-up of this cohort was performed in 2008. At both occasions the questionnaire was mailed in springtime. Swedish personal identification numbers were used to get updated addresses from the Swedish Population Address Register (SPAR). Addresses were found for 2,403 of the original 2,572 participants (Figure 1); 169 were unreachable: 106 had personal identification numbers not matching the SPAR register, 35 had emigrated, 21 had moved without providing a forwarding address, five were deceased, and two were not traceable for reasons of secrecy.

### Questionnaire

In 1995 the questionnaire was based on the Toulihampi questionnaire [17]. The questionnaire in 2008 was based on the Nordic Occupational Skin Questionnaire 2002 (NOSQ-2002), [18]. The questions regarding hand eczema were almost the same in the two questionnaires and the answer alternatives were exactly the same. Some additional questions constructed by the investigators were included in the 2008 questionnaire (See Additional file 1).

Topics surveyed by the questionnaire were: hand eczema, childhood eczema, asthma and rhino-conjunctivitis, household size and family structure, occupation and everyday activities, hand washing and skin care.

### Distribution of the questionnaire

A self-administrated postal questionnaire and a pre-paid return envelope were distributed in late May 2008. A postcard was sent at the beginning of June as a first reminder. At the end of August, a second reminder was sent which included a copy of the questionnaire, a pencil and a pre-paid return envelope. Finally, a postcard was sent in the middle of September as a third and final reminder.

### Data analysis and statistics

One-year prevalence of hand eczema was estimated from reported hand eczema at present or having had hand eczema some time during the last 12 months (See Additional file 1). The question regarding the 1-year prevalence was previously validated [19,20]. The question on point prevalence was validated, and sensitivity (73%) and specificity (99%) were calculated [15]. To estimate the true 1-year prevalence for this cohort, a calculation of the 1-year prevalence in relation to sensitivity and specificity was made by using the formula: P = (P * + (specificity–1))/(sensitivity + (specificity–1)). P is the estimated true 1-year prevalence in the population and P* is the 1-year prevalence in the sample [5,15,21].

The cumulative incidence was calculated on the individuals reporting having 1-year prevalence or ever having had hand eczema 2008 minus those who had 1-year prevalence or ever had had hand eczema in 1995. The cumulative incidence is presented as the percentage of new cases of hand eczema in the cohort. Incidence rate is presented as new cases per 1000 person-years, i. e. the cumulative incidence/13 years × 1000.

Four groups were constructed with the intention to analyse risk factors and the development of hand eczema over time. The groups were constructed as follows: those who reported having a 1-year-prevalence in 1995 and in 2008 are in group HX9508, those who reported having a 1-year-prevalence in 1995 but not in 2008 are in group HX95, those who reported having a 1-year-prevalence in 2008 but not in 1995 are in group HX08, and those who reported that they never had hand eczema are in group NoHX.

The reliability over time of self-reported childhood eczema in 1995 and then reporting the same in 2008 was determined by calculating positive predictive value (PPV); i.e. the percentage positive agreement in 2008 among the yes-respondents from 1995. The negative predictive value (NPV); i.e. the agreement of no-answers in 1995 and 2008 was also calculated.

Potential exogenous risk factors for developing hand eczema such as household size, time required for household work, frequency of hand washing, skin protective habits, working hours outside home and leisure activities were investigated by dividing the cohort into two groups. The respondents who had 1-year prevalence of hand eczema 2008, i.e. the merged groups HX9508 and HX08, denominated the HX group, and the group that reported never having had hand eczema, the NoHX group. Furthermore, hand eczema was also studied in the two hand eczema groups separately regarding these factors.

Regarding occupation, the respondents were asked not only to tell their profession, but also to give information about work tasks.

The groups HX9508, HX95 and HX08 were compared to the group NoHX using a multinominal logistic regression model. The endogenous factors childhood eczema, asthma and rhino-conjunctivitis as reported in 2008 were used. The response choices in this calculation were Yes/No. Exogenous factors such as hand-washing (times a day), usage of moisturisers (dichotomised Daily/Some time each week, some time each month, never), cooking, cleaning/washing laundry, and taking care of children 0–4 years of age (hours a day) were investigated.

Categorical data were presented as numbers and/or proportions in groups; quantitative data were presented by mean, median and quartiles. Nominal data were tested with the Chi-squared test. When the number of expected values was insufficient, Fisher’s exact test was used. When comparing groups over time, McNemar’s test was used. Ordinal and interval data were tested with Kruskal-Wallis H test and Mann–Whitney U-test in independent group comparisons. In the multinominal logistic regression analysis odds-ratios, 95% confidence intervals and p-values were given for all the covariates. If data was missing for any covariate, the individual was not included in the analysis. A p-value <0.05 was considered significant in all calculations. All statistical analyses were performed with SPSS 20.0 for Windows.

### Ethics

The study was approved by the The Regional Ethical Review Board in Lund, (application no 156/2008).

## Results

The flow-chart of the cohort is shown in Figure 1. Out of the 2,403 participants from the original cohort who received a questionnaire in the mail, 1,516 responded to the questionnaire, which was a response rate of 63%; 56% of the respondents were females. Significantly more females than males answered the questionnaire, 69.4% of the reachable original female cohort and 56.4% of the males (p < 0.001). However, in 2008 there were no significant differences between the respondents and non-respondents in reporting 1-year prevalence of hand eczema in 1995 (p = 0.677). No significant differences were found within the genders in reported hand eczema in 1995 (females, p = 0.490; males, p = 0.297).

In the first dispatch, 899 (37%) responded, the first postcard reminder yielded 158 (10%) responses. On the second reminder 437 (32%) responded. With the final postcard reminder, 22 (2%) responded, which left 887 non-respondents.

### One-year prevalence of hand eczema

**Figure 2:**
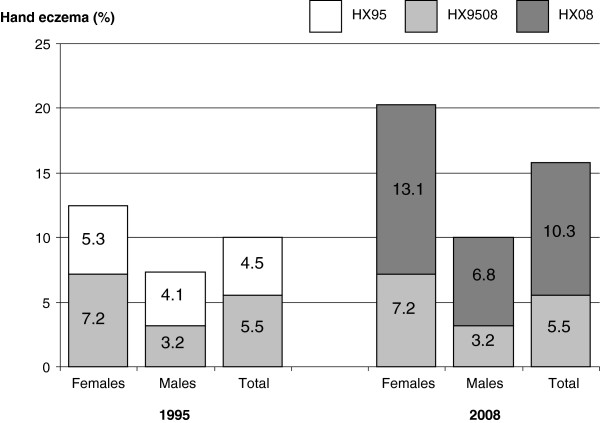
The proportions reporting hand eczema in 1995 but not 2008 (HX95), both 1995 and 2008 (HX9508), and only 2008 (HX08).

The 1-year prevalence of hand eczema in 2008 was 15.8%, Figure 2; females reported hand eczema twice as often as males, 20.3% versus 10.0%, (p < 0.001). The estimated true 1-year prevalence for this cohort was: (0.158 + (0.99 – 1)) / (0.73 + (0.99 – 1)) = 20.6%, 26.8% for females and 12.5% for males.The 1516 participants were allocated to any of the four groups as previously defined; HX9508 (83/1516, 5.5%, 7.2% females and 3.2% males), HX95 (71/1516, 4.7%; 5.6% females and 3.5% males), HX08 (157/1516, 10.4%; 13.1% females and 6.8% males) and NoHX (1016/1516, 67.0%; 61.4% females and 74.4% males). One hundred and sixty respondents (10.6%) reported that they had had hand eczema at some time, but not in 1995 nor in 2008, 29 individuals, 1.9%, did not answer the question. The higher proportion of females compared with males in the hand eczema groups compared with the NoHX group was significant (p < 0.001).

### Incidence of hand eczema

In 1995 in total 13.3% (202/1516) reported they had or had had hand eczema, 139 females, (16.2%) and 63 males (9.6%), p < 0.001. In 2008 an additional 198 individuals reported themselves having or having had hand eczema. Thus the cumulative incidence over the 13 years was 15.1% (198/1314), for the females 18.6% and for the males 10.7%, p < 0.001. The incidence rate was estimated as 11.6 cases per 1000 person-years, 14.3 for females and 5.2 for males (p < 0.001).

### Hand eczema versus childhood eczema, asthma, rhino-conjunctivitis and gender

Childhood eczema was reported by 400/1516 (26.4%) of the participants. The proportions of having had childhood eczema, asthma and rhino-conjunctivitis in the four groups in total and by gender for 2008 are shown in Table 1. The proportions of the individuals reporting only childhood eczema; i.e. not in combination with asthma and/or rhino-conjunctivitis (146/1516, 9.6%), were found to be: HX9508, 73.9%; HX95, 41.7%, HX08, 45.5% and NoHX, 17.3% (p < 0.001). Only having had asthma was reported by 22/1516 (1.5%); within the groups: 1, 1, 0 and 20 individuals respectively, (p = 0.366). Only having had rhino-conjunctivitis was reported by 201/1516 (13.3%). Within the groups 4, 7, 11 and 179 individuals, respectively (p = 0.124).

**Table 1:** Prevalence of self-reported childhood eczema and/or asthma and/or rhino-conjunctivitis in 2008 with respect to 1-year prevalence of hand eczema and gender in the groups HX9508 (1-year prevalence of hand eczema 1995 and 2008), HX95 (1-year prevalence of hand eczema only 1995), HX08 (1-year prevalence of hand eczema only 2008) and NoHX (never having had hand eczema)

**The 2008 questionnaire**	**Group HX9508**			**Group HX95**			**Group HX08**			**Group NoHX**		
**“Did you have eczema in your childhood?” (n = 1325, 100%)**	**Females n (%)**	**Males n (%)**	**Total n (%)**	**Females n (%)**	**Males n (%)**	**Total n (%)**	**Females n (%)**	**Males n (%)**	**Total n (%)**	**Females n (%)**	**Males n (%)**	**Total n (%)**
“No” (n = 792, 59.8%)	10 (16.1)	3 (14.3)	13 (15.7)	19 (39.6)	6 (27.3)	25 (35.7)	38 (33.9)	14 (31.1)	52 (33.1)	349 (66.5)	353 (72.0)	702 (69.2)
“Yes” (n = 400, 30.2%)	48 (77.4) **a>**	15 (71.4) **a>**	63 (75.9) **b>**	22 (45.8) **a>**	14 (63.6) **a>**	36 (51.4) **b>**	63 (56.3) **a>**	23 (51.1) **a>**	86 (54.8) **b>**	140 (26.7) **a<, c>**	75 (15.3) **a<**	215 (21.2) **b<**
“I do not know” (n = 133, 10.0%)	4 (6.5)	3 (14.3)	7 (8.4)	7 (14.6)	2 (9.1)	9 (12.9)	11 (9.8)	8 (17.8)	19 (12.1)	36 (6.9)	62 (12.7)	98 (6.6)
**“Have you ever had asthma?”(n = 1326)**												
“No” (n = 1082, 81.6%)	37 (59.7)	15 (71.4)	52 (62.7)	38 (79.2)	19 (86.4)	57 (81.4)	84 (75.0)	35 (77.8)	119 (75.8)	433 (82.3)	421 (85.9)	854 (84.0)
“Yes” (n = 217, 16.4%)	23 (37.1)	6 (28.6)	29 (34.9) **b>**	8 (16.7)	2 (9.1)	10 (14.3) **b<**	23 (20.5)	10 (22.2)	33 (21.0) **b>**	84 (16.0)	61 (12.4)	145 (14.3) **b<**
“I do not know” (n = 27, 2.0%)	2 (3.2)	0	2 (2.4)	2 (4.2)	1 (4.5)	3 (4.3)	5 (4.5)	0	5 (3.2)	9 (1.7)	8 (1.6)	17 (1.7)
**“Have you ever had allergic symptoms from your nose or eyes?“ (n = 1306)**												
“No” (n = 664, 50.8%)	20 (32.3)	5 (25.0)	25 (30.5)	26 (54.2)	11 (52.4)	37 (53.6)	41 (36.9)	16 (36.4)	57 (36.3)	280 (54.2)	265 (54.9)	545 (54.5)
“Yes” (n = 582, 44.6%)	40 (64.5) **a>**	15 (75.0) **a>**	55 (67.1) **b>**	21 (43.8) **a<**	10 (47.6) **a<**	31 (44.9) **b<**	63 (56.8) **a<**	24 (54.5) **a<**	87 (55.4) **b>**	211 (40.8) **a<**	198 (41.0) **a<**	409 (40.9) **b<**
“I do not know” (n = 60, 4.6%)	2 (3.2)	0	2 (2.4)	1 (2.1)	0	1 (1.5)	7 (6.3)	4 (9.1)	13 (8.3)	26 (5.0)	20 (4.1)	46 (4.6)

Significant differences (p < 0.05) between groups, totals and/or genders are **marked with bold letters**. **a**: significant differences within females or within males in different groups, **b:** significant difference between totals, **c**: significant difference between females and males in a group, **<** or **>**: the group or the gender has significantly lower or significantly higher frequency than the compared group. Chi-squared test.

### Self-reported childhood eczema in 2008 compared to 1995

The question about childhood eczema was answered by 1323 of the 1516 respondents (87.3%) in 2008. In 1995, 297/1323 individuals (22.4%) reported childhood eczema, and 239 of these gave the same answer in 2008. This gives the positive predictive value (PPV) of 80.5% (239/297). The negative predictive value (NPV), i.e. reporting not having had childhood eczema in 1995 as well as in 2008, was 76.7% (610/795). When comparing genders, the PPV for females was 82.3% and the NPV was 77.0%. The PPV for males was 75.6% and the NPV was 76.5%. There were significant differences within three of the four groups between PPV and NPV; HX9508 group: PPV = 90.6% and NPV = 35.0% (p = 0.016); HX95 group: PPV = 76.7% and NPV = 60.7% (p = 0.611); HX08 group: PPV = 94.0% and NPV = 55.3% (p < 0.001); NoHX group: PPV = 73.8% and NPV = 77.6% (p < 0.001).

### Hand eczema and exogenous factors

The results regarding potential exogenous risk factors for developing hand eczema are shown in Table 2. The individuals in the HX group reported a significantly higher frequency of hand washing compared to the NoHX group, mean 15.4 versus 11.7 times per day (p < 0.001). The females in the HX group had a significantly higher number of daily hand washing compared to the females in the NoHX-group, 17.4 versus 14.5 times per day (p < 0.001).

**Table 2:** Comparisons of exogenous factors between the group with a 1-year prevalence of hand eczema in 2008 (Group HX), and the group reporting never having had hand eczema (Group NoHX)

	**Group HX**			**Group NoHX**		
	**Mean, Median, (Q1 – Q3)**			**Mean, Median, (Q1 – Q3)**		
	**Females**	**Males**	**Total**	**Females**	**Males**	**Total**
Number of persons in the household, yourself included (n = 1254)	3.0, 3, (2 – 4) **a>, c>**	2.5, 2, (2 – 3)	2.8, 3, (2 – 4) **b>**	2.7, 3, (2 – 4) **c>**	2.4, 2, (1 – 3)	2.6, 2, (2 – 4)
Number of children below 4 years of age (n = 1191)	0.8, 1, (0 – 1) **c>**	0.5, 0, (0 – 1)	0.7, 1, (0 – 1)	0.7, 0, (0 – 1) **c>**	0.6, 0, (0 – 1)	0.6, 0, (0 – 1)
Hours a day taking care of children 0 – 4 y (n = 1165)	5.3, 3, (0 – 8) **c>**	1.6, 0, (0 – 3)	4.3, 1, (0 – 6) **b>**	5.1, 0, (0 – 6) **c>**	2.0, 0, (0 – 3)	3.6, 0, (0 – 5)
Hours a day cooking (n = 1245)	1.3, 1, (1 – 1.5) **c>**	1.2, 1, (1 – 1)	1.2, 1, (1 – 1) **b>**	1.3, 1, (1 – 1.5) **c>**	1.0, 1, (0.5 – 1)	1.1, 1, (1 – 1)
Hours a day cleaning/making laundry (n = 1236)	1.3, 1, (1 – 2) **a>, c>**	0.7, 1, (0.3 – 1)	1.2, 1, (1 –1) **b>**	1.1, 1, (1 – 1) **c>**	0.7, 1, (0.2 – 1)	0.9, 1, (0.5 – 1)
Number of times a day washing hands at home (n = 1241)	8.8, 7, (5 – 10) **a>, c>**	4.4, 3.5, (3 - 5)	7.6, 6, (4 – 10) **b>**	7.2, 5, (4 – 10) **c>**	4.4, 4, (3 – 5)	5.9, 5, (3 – 7)
Number of times a day washing hands at work (n = 1193)	9.2, 6, (4 – 10) **a>, c>**	6.2, 3.5, (3 - 8) **a>**	8.3, 5, (3 – 10) **b>**	7.5, 5, (3 – 10) **c>**	4.5, 3, (2 – 5)	6.0, 4, (3 – 6)
Number of times a day washing hands, at home and at work (n = 1189)	17.4, 13.3, (10–20)**a>, c>**	10.6, 8 (5.8 - 14)	15.4, 12, (8 –17.8) **b>**	14.5, 11, (8 – 15) **c>**	8.8, 7 (5 – 10)	11.7, 9, (6 – 14)
If smoking; number of cigarettes a day (n = 112)	9.6, 8, (3.5 – 15)	7.3, 5, (2 – 15)	9.3, 8,(3.3 – 15)	6.5, 5, (2 - 10)	7.6, 5.5, (2 – 11.5)	7.1, 5, (2 – 10)
If using protective gloves at work: hours a day using them (n = 398)	2.8, 2, (1 – 3) **c<**	3.5, 3, (1.5 – 5.5)	2.9, 2, (1 – 4)	2.3, 2, (1 - 3)	3.8, 2, (1 – 6)	3.1, 2, (1 – 4)
Number of working hours at ordinary work (n = 1212)	35.6, 40, (30 – 40) **c<**	41.7, 40, (40 – 45)	37.3, 40, (34 – 40) **b<**	36.7, 40, (34 - 40)	41.9, 40, (40 – 45)	39.2, 40, (38 – 40)
Number of working hours at additional work (n = 107)	4.7, 3, (2 – 7.3)	11.2, 12.5(1.5 -20)	6.1, 3.5, (2 – 8)	10.4, 5, (2 – 12)	9.0, 6, (3 – 10)	9.6, 5, (3 – 10)
Number of working hours at ordinary and additional work (n = 101)	39.8, 41, (30 – 48) **c<**	51.0, 56,(41–59)	42.0, 42.5,(31-51)	44.3, 43 (39 – 50)	50.6, 50, (44 – 55)	47.9, 46, (41 – 53)
Hours a week gardening (during summer season). (n = 1201)	2.3, 1, (0 – 3)	2.5, 1, (0 – 3)	2.4, 1, (0 – 3)	2.5, 1, (0 – 3)	2.7, 1, (0 – 3)	2.6, 1, (0 – 3)
Hours a week repairing cars/engines (n = 1168)	0.2, 0, (0 – 0) **c<**	2.9, 0, (0 – 1)	1.0, 0, (0 – 0)	0.1, 0, (0 – 0)	1.5, 0, (0 – 1)	0.8, 0, (0 – 0)
Hours a week doing building work, restoration (n = 1179)	2.4, 0, (0 – 1) **c<**	3.6, 1, (0 – 3)	2.7, 0, (0 – 2)	2.4, 0, (0 – 1) **c<**	5.1, 1, (0 – 4.75)	3.8, 0, (0 – 2)
Hours a week doing sports/athletics (n = 1184)	4.4, 2, (1 – 4)	3.3, 2, (1 – 4)	4.1, 2, (1 – 4)	4.0, 2, (1 – 5)	4.2, 2, (1 – 4)	4.1, 2, (1 – 4)
Hours a week doing hobbies (n = 979)	4.3, 2, (0 – 5) **c<**	4.6, 2, (0 – 4.5)	4.4, 2, (0 – 5)	3.4, 2, (0 – 4.5)	5.2, 2, (0 – 5)	4.3, 2, (0 – 5)

Significant differences (p < 0.05) between groups, totals and/or genders are **marked with bold letters**. **a**: significant differences within females or within males in different groups, **b:** significant difference between totals, **c**: significant difference between females and males in a group, **<** or **>**: the group or the gender has significantly lower or significantly higher frequency than the compared group. Mann-Whitney U test.

Concerning skin care, daily use of moisturisers was reported by 60.5% in the HX group (females 67.6% males 41.5%), and by 30.6% in the NoHX group (females 47.4% and males 12.7%). The differences were significant between the two groups and between the genders within the groups (p < 0.001). Regardless of hand eczema, females used moisturisers significantly more often than males; 52.9% female versus 16.2% male daily users (p < 0.001), However, having hand eczema raised the reported usage of moisturizers by a factor 1.4 for females and 3.3 for males.

The exogenous factors were analysed between all four groups, in total as well as between genders (HX9508, HX95, HX08 and NoHX) and within genders in all groups, Table 3. In total as well as within females, the HX08 group had a significantly higher frequency of hand washing at home and at work than the NoHX group (p < 0.001). Regarding time spent at ordinary work; the HX08 group worked significantly less than the NoHX group (p = 0.001). The HX08 group spent significantly more time cooking, cleaning and doing laundry than the NoHX group. The HX08 group smoked significantly more cigarettes than those in the HX9508 and NoHX groups (p = 0.023 and 0.012 respectively).

**Table 3:** Comparisons of exogenous factors between the group HX9508, i.e. having had 1-year prevalence of hand eczema 1995 and 2008, the group HX95, i. e. having had hand eczema only 1995, the group HX08, i.e. having eczema only 2008 and the group NoHX, i. e. the group reporting never having had hand eczema

	**HX9508**			**HX95**			**HX08**			**NoHX**		
	**Mean (Q1-Q3)**			**Mean (Q1-Q3)**			**Mean (Q1-Q3)**			**Mean (Q1-Q3)**		
	**Females**	**Males**	**Total**	**Females**	**Males**	**Total**	**Females**	**Males**	**Total**	**Females**	**Males**	**Total**
Number of persons in the household, yourself included (n = 1324)	2.9 (2-4)	2.5 (1-3)	2.8 (2-4)	2.9 (2-4)	2.6 (2-4)	2.8 (2-4)	3.0 (2-4) **a>, c>**	2.4 (2-3)	2.9 (2-4) **b>**	2.7 (2-4) **a<, c<**	2.4 (1-3)	2.6 (2-4) **b<**
Number of children below 4 years of age (n = 1259)	0.8 (0-1)	0.7 (0-1)	0.7 (0-1)	0.6 (0-1)	0.5 (0-1)	0.6 (0-1)	0.8 (0-1) **a>**	0.5 (0-1)	0.7 (0-1)	0.7 (0-1) **a<**	0.6 (0-1)	0.6 (0-1)
Hours a day taking care of children 0 – 4 years of age (n = 1234)	4.9 (0-8) **a<**	1.9 (0-3)	4.2 (0-6)	5.8 (0-8) **a>**	1.0 (0-1.5)	4.3 (0-5)	5.5 (0-8) **a>**	1.5 (0-2)	4.4 (0-6.3)	5.1 (0-6) **a>**	2.0 (0-3)	3.6 (0-5)
Hours a day cooking (n = 1314)	1.2 (1-1.3)	1.2 (1-1)	1.2 (1-1)	1.3 (1-2) **c>**	0.9 (0.5-1)	1.2 (1-1)	1.3 (1-2) **c>**	1.2 (0.8-1)	1.3 (1-1.3) **b>**	1.3 (1-1.5) **c>**	1.0 (0.5-1)	1.1 (1-1) **b<**
Hours a day cleaning/making laundry (n = 1304)	1.3 (1-1.6) **c>**	0.8 (0.3-1)	1.2 (1-1) **b>**	1.1 (1-1) **c>**	0.7 (0.4-1)	1.0 (1-1)	1.3 (1-2) **a>, c>**	0.7 (0.4-1)	1.2 (1-1) **b>**	1.1 (1-1) **a<, c>**	0.7 (0.2-1)	0.9 (0,5-1) **b<**
Number of times a day washing hands at home (n = 1309)	7.8 (4-10) **c>**	3.8 (2-5)	6.8 (3-10)	6.3 (5-8) **a<, c>**	5.0 (3-5.3)	5.9 (4-8) **b<**	9.3 (5-10) **a>, c>**	4.7 (3-5)	8.0 (4-10) **b>**	7.2 (4-10) **a<, c>**	4.4 (3-5)	5.9 (3-7) **b<**
Number of times a day washing hands at work (n = 1260)	8.3 (4-10) **c>**	5.2 (2.5-4)	7.4 (3 –10) **b>**	7.6 (3.5-11) **c>**	4.3 (3-4)	6.5 (3-8)	9.7 (4-10) **a>, c>**	6.6 (3-10) **a>**	8.7 (3-10) **b>**	7.5 (3-10) **a > c<**	4.5 (2-5) **a<**	6.0 (3-6) **b<**
Number of times a day washing hands, at home and at work (n = 1255)	14.9 (9-17) **c>**	9.0 (5-11.5)	13.3 (7-16)	14.0 (9-19.5) **c>**	9.3 (6.8-10)	12.4 (8-16) **b<**	18.7 (10-21.5) **a>, c>**	11.3 (6-15) **a>**	16.5 (8-20) **b<**	14.5 (8-16) **a > c<**	8.8 (5-10) **a>**	11.7 (6-14) **b<**
If smoking; number of cigarettes a day (n = 112)	7.5 (5.8.7.8)	3.5 (2-5)	6.7 (2.9.7.3) **b<**	8.1 (5-11.8)	1 (1.0)	7.7 (4.5– 10.5)	10.9 (6-15) **a>**	15 (1.0)	11.1 (7-15) **b>**	6.5 (2-10) **a<**	7.6 (2-11.5)	7.1 (2-10) **b<**
If using protective gloves at work: hours a day using them (n = 398)	2.8 (13-.5)	3.9 (2-5.8)	3.1 (1.5-4.5) **b>**	1.6 (1-2)	2.4 (0.5-4.5)	1.8 (1 – 2.8) **b<**	2.8 (1-3)	3.2 (1-5.5)	2.9 (1-3.3)	2.3 (1-3) **c<**	3.8 (1-6)	3.1 (1-4)
Number of working hours at ordinary work (n = 1279)	38.3 (35 – 40.5) **a>**	41.7 (40-44)	39.2 (38-42) **b>**	35.5 (32-40) **c<**	44.7 (40-50)	38.5 (36 – 45)	34.1 (30-40) **a<, c<**	41.8 (40-45)	36.3 (30-40) **b<**	36.7 (32-40) **a>, c<**	41.9 (40-45)	39.2 (38-40) **b>**
Number of working hours at ordinary and additional work (n = 107)	42.7 (32-49)	57.0 (57.0)	44.1 (33-51.8)	33.5 (29-38)	49.5 (45-54.5)	44.2 (35.8,51.5)	37.7 (29.3,47.3)	49.5 (39-59)	40.7 (30.5,51.8)	44.3 (38.5,49.5) **c<**	50.6 (44-55)	47.9 (41-53)
Hours a week gardening (n = 1270)	2.1 (0-3)	1.9 (0-3.5)	2.1 (0-3)	3.5 (0-3)	1.4 (0-2)	2.8 (0 – 2.5)	2.4 (0-2.3)	2.8 (0-3)	2.5 (0-2.5)	2.5 (0-3)	2.7 (0-3)	2.6 (0-3)
Hours a week repairing cars/engines (n = 1236)	0.2 (0-0)**<**	3.6 (0-1)	1.0 (0 -0)	0.1 (0-0) **c<**	0.5 (0-1)	0.2 (0-0)	0.3 (0-0) **c<**	2.6 (0-1)	1.0 (0-0)	0.1 (0-0) **c<**	1.5 (0-1)	0.8 (0-0)
Hours a week doing building work, restoration (n = 1245)	1.7 (0-2) **c<**	4.2 (0.6-5)	2.3 (0-2)	1.1 (0-1.5)	3.1 (0-3.5)	1.7 (0-2)	2.8 (0-1)	3.3 (0-2)	2.9 (0-1.5)	2.4 (0-1) **c<**	5.1 (0-4.8)	3.8 (0-2)
Hours a week doing sports (n = 1251)	4.2 (1-4)	2.7 (1-3.7)	3.8 (1-4)	2.5 (1-4)	2.7 (1-3.3)	2.6 (1-4)	4.5 (1-4.8)	3.6 (0.5-4)	4.2 (1-4)	4.0 (1-5)	4.2 (1-4)	4.1 (1-4)
Hours a week doing hobbies (n = 1035)	4.7 (2-5)	3.4 (0-6)	4.4 (2-5)	3.2 (0-4.8)	1.8 (0-3)	2.7 (0-4)	4.0 (0-4)	5.0 (0-4)	4.3 (0-4)	3.4 (0-4.5) **c<**	5.2 (0-5)	4.3 (0-5)

Significant differences (p < 0.05) between groups, totals and/or genders are **marked with bold letters**. **a**: significant differences within females or within males in different groups, **b:** significant difference between totals, **c**: significant difference between females and males in a group, **<** or **>**: the group or the gender has significantly lower or significantly higher frequency than the compared group/groups. Kruskal-Wallis Test and Mann-Whitney U test.

Among the respondents 487/1323 (36.8%) used moisturisers daily. The HX9508 group used moisturisers significantly more than the other groups, 71.1%, followed by the HX08 group, 54.8%, the HX95group, 45.7% and the NoHX group, 30.6%, (p < 0.001). Among females 52.7% (n = 746), used moisturisers every day; 79% in the HX9508 group, 61.3% in the HX08 group, 56.2% in the HX95 group and 47.4% in the NoHX group (p < 0.001). Among males 16.3% used moisturisers daily: 47.6% in the HX9508 group, 38.6% in the HX08 group, 22.7% in the HX95 group and 12.7% in the NoHX group (p < 0.001). Males *with* hand eczema used moisturisers as often as women *without* hand eczema.

### Factors predicting hand eczema

The analysis of endogenous and exogenous factors was performed with multinominal logistic regression. The results are shown in Table 4. Having had childhood eczema was the most significant predictor for 1-year prevalence of hand eczema 2008 with odds ratios of 13.17 in the group HX9508 and 5.17 in the group HX08 compared to the group NoHX. The frequency of daily hand washing was significantly associated with the 1-year prevalence of hand eczema only in the HX08 group. The daily usage of moisturisers was significantly associated with 1-year prevalence of hand eczema in the groups HX9508 and HX08. High odds ratios, 1.40, for predicting 1-year prevalence of hand eczema was found for female gender in the group HX9508. In the group HX08 the higher odds ratio for females was 1.19. However, none of these differences were significant.

**Table 4:** Endogenous and exogenous factors associated with hand eczema analysed with logistic multinominal regression method, Group NoHX: never having had hand eczema, Group HX9508: having hand eczema 1995 as well as 2008, Group HX95: having had hand eczema only 1995 and Group HX08: having hand eczema only 2008

**Group**	**Group HX9508 vs Group NoHX (N = 852)**		**Group HX95 vs Group NoHX (N = 836)**		**Group HX08 vs Group NoHX (N = 895)**	
	**Odds-ratio**	**95% CI for OR (p-value)**	**Odds-ratio**	**95% CI for OR (p-value)**	**Odds-ratio**	**95% CI for OR (p-value)**
Having had childhood eczema	**13.17**	**6.74 – 25.72 (<0.001)**	**4.12**	**2.31 – 7.33 (<0.001)**	**5.17**	**3.33 – 8.03 (<0.001)**
Having had asthma	1.89	0.99 – 3.62 (0.54)	0.81	0.34 – 1.89 (0.619)	1.12	0.64 – 1.94 (0.699)
Having had rhino-conjunctivitis	1.64	0.86 – 3.10 (0.132)	0.98	0.53 – 1.81 (0.945)	1.51	0.95 – 2.40 (0.084)
Female gender	1.40	0.71 – 2.75 (0.334)	1.42	0.73 – 2.79 (0.304)	1.19	0.72 – 1.97 (0.500)
Number of times a day washing hands, at home and at work	0.99	0.97 – 1.02 (0.696)	1.00	0.97 – 1.03 (0.858)	**1.02**	**1.01 – 1.04 (0.038)**
Usage of moisturisers: daily vs less than daily	**5.17**	**2.82 – 9.51 (<0.001)**	1.49	0.81 – 2.73 (0.199)	**2.11**	**1.34 – 3.30 (0.001)**
Cooking:hours a day	1.00	0.69 – 1.43 (0.987)	1.00	0.66 – 1.51 (0.997)	1.10	0.87 – 1.37 (0.433)
Washing and cleaning: hours a day	1.19	0.81 – 1.77 (0.377)	0.81	0.48 – 1.39 (0.446)	1.23	0.94 – 1.60 (0.126)
Taking care of children < 4 years old: hours a day	1.01	0.97 – 1.06 (0.616)	1.02	0.98 – 1.07 (0.321)	0.99	0.96 – 1.03 (0.707)

Odds-ratios (OR) in predicting 1-year prevalence of hand eczema 2008 compared to the group NoHX, group HX9508 compared to group NoHX and group HX08 compared to group NoHX . Confidence intervals (CI), 95%, and p-values are given for all variables. **Significant OR, CI and p-values are in bold text.**

## Discussion

In this study comprising 1,516 young adults, the 1-year prevalence of hand eczema was more than 15%. One third of these individuals also had 1-year prevalence at the baseline 1995. The 1-year prevalence, and not the point prevalence, was used in all calculations because it better reflects the persistency, the relapsing course and the seasonal variations of the disease [2,19]. The increase in the one-year prevalence between the two occasions is in accordance with previous large Swedish cross-sectional studies with respect to the age groups [3-5,22].

The estimated incidence of hand eczema in our study was 11.6 cases per 1000 person-years, 14.3 among females and 5.2 among males. Our figures are in the upper amplitude compared to an earlier population based study from Sweden, which showed between 11.4 and 3.7 cases/1000 person-years among 20–29 year-old females and males, respectively [23]. One explanation could be that our study is prospective, and underreporting is to be expected in retrospective questionnaire studies [24]. Based on 7 European hand eczema studies performed among 16–77 years-olds, the median incidence rate of hand eczema was 9.6 cases/1000 person-years (range 4.6–11.4) among women and 4.0 cases/1000 person-years (range 1.4–7.4) among men [1], which is also slightly lower than our current findings, probably due to age-differences. To the best of our knowledge there are no comparable studies of the cumulative incidence in this age group. The cumulative incidence of hand eczema in our study across 13 years was 15.1% (18.6% for females and 10.7% for males). This can be considered to be a high proportion [15]. When using a questionnaire for estimating the true occurrence of a disease it is important to know the sensitivity and specificity of the question used. The question on 1-year prevalence of hand eczema underestimates the occurrence. [25]. However, regarding childhood eczema the occurrence has been found to be overestimated especially if the true prevalence is low [5,19]. Based on prevalence as well as incidence, the occurrence of hand eczema is approximately twice as common among females compared to men, which is similar to other population-based studies [1,26,27].

The advantage of a longitudinal cohort study compared with a cross-sectional study is that it enables the estimation of both cumulative incidence and incidence rate. Another advantage of performing a follow-up study is the possibility to compare the development of hand eczema over time in relation to different risk factors.

The four groups (HX9508, HX95, HX08 and NoHX) were used to investigate the relationship between childhood eczema and the incidence of hand eczema. The assumption was that a smaller proportion of individuals who had hand eczema in 2008 but not in 1995 reported childhood eczema. However, there were no significant differences between the three hand eczema groups concerning childhood eczema. Furthermore, it was found that a higher proportion of individuals who had hand eczema at both occasions reported childhood eczema.

Thus, in this cohort childhood eczema was the most important predicting factor regardless of the debut of hand eczema. In 2008, around 30% of our sample reported childhood eczema (females 36%, males 20%). In a large population-based Swedish study performed from 2002–2003, among 21–30 years-olds, childhood eczema was reported by 30.1% of females and 20.8% of males, [4,28,29]. The corresponding figures in the 31–40 year-olds were 21.8% and 16.2% [30]. Thus, in our study, the prevalence of childhood eczema was higher. Similar to other studies, the relationship between having had hand eczema and reporting childhood eczema was highly significant [31]. The agreement in self-reports of childhood eczema at the two occasions was high. This high reliability over time in this age-group can be useful to know when hand eczema is diagnosed. However, the lower rate of reported childhood eczema in 2008 can be explained by recall bias as was found in a study comprising respondents aged 31 to 42 years [32]. For the individuals who reported only rhino-conjunctivitis, there was no significant association with one-year prevalence of hand eczema. Also, there was no association with asthma only, but there were very few respondents. Thus in our study no additional information concerning risk for hand eczema was obtained by asking about asthma or rhino-conjunctivitis. These results are in accordance with Meding et al. who showed that asthma and rhino-conjuntivitis in adults were only associated with hand eczema at an age below 30 years [23]; in another study, including adolescents, a marginally significant association with inhalant allergy was found [33].

Analyses of exogenous factors showed that the individuals with hand eczema only in 2008, reported a significantly higher frequency of hand washing compared to the individuals without hand eczema.

Females with hand eczema spent significantly more time doing household activities than men with hand eczema (Table 3). Hand washing was more frequent among females with hand eczema than females without hand eczema as well as compared with men with hand eczema. In the multinominal regression analyses hand washing in the group HX08 was the only significant exogenous risk factor associated with hand eczema. In the majority of hand eczema studies hand washing is found to be the most significant risk factor for developing hand eczema [34]. In our cohort, other exogenous risk factors such as cooking, washing and cleaning and taking care of young children did not have any significant association with hand eczema. Furthermore, female gender was not a significant risk factor. However, it is well known that females have hand eczema more often than men. This can be explained by the high exposure to water and other skin irritants. Experimental as well as epidemiological studies [14,35] have demonstrated that female skin is not more sensitive to irritants than male skin [35] which is in line with our findings.

An interesting finding was the high odds-ratio in daily use of moisturisers in the two groups with current 1-year prevalence of hand eczema (HX9508 and HX08). This pattern was not seen in the group having had hand eczema in 1995 (HX95).

When self-administrated questionnaires are used, it is important for the results to be adjusted based on sensitivity and specificity of validated questions. This is especially important in diseases that are common and affect the general health and well-being of individuals, such as hand eczema. The development of specific instruments like questionnaires implicates problems. In this case the questions regarding childhood and hand eczema were not validated in 1995 but 2,535 of the 2,572 pupils (98.6%) were clinically examined, and the sensitivity of 73% and the specificity of 99% were found [15]. The question regarding the 1-year prevalence of hand eczema, which was used in the present study and in the first study, was previously validated [19]. Thus, the true one-year prevalence of hand eczema can be estimated from our data and is 20.6% for all; 26.8% among females and 12.5% among males.

The answers to the open questions on occupation as well as work tasks gave no further information regarding risk factors for developing or maintaining hand eczema. This circumstance seems to be a common problem in questionnaire studies [3]. In a study regarding occupational exposure to water as a risk factor for hand eczema, it was found that the title of an occupation gave misclassified results; exposure time and frequency of water use were more appropriate measures [36]. For result validity, it is important to have high response rates in general population studies [37-39]. The response rate in this study was almost two thirds of the individuals who received a questionnaire in the mail. Females were significantly more willing to participate than the males. There were, however, no significant differences within the female or the male groups regarding having had 1-year prevalence of hand eczema at the two occasions. The response rate was similar to the annual national public health questionnaire performed by Swedish National Institute of Public Health [40].

## Conclusions

This study demonstrated that incidence of hand eczema in early adulthood tends to be associated with factors in everyday life such as frequent hand-washing. Regarding childhood eczema, the odds ratio for having hand eczema was twice as high in the HX9508 group compared to the group HX08, indicating a high vulnerability in this group. Furthermore, early onset of hand eczema seemed to be related to endogenous risk factors such as a history of childhood eczema. The higher frequency of hand eczema among women depended on exogenous factors.

## Competing interests

The authors declare that they have no competing interests.

## Authors’ contributions

The authors together designed the study, analysed the data and wrote the manuscript. All authors read and approved the final manuscript.

## Pre-publication history

The pre-publication history for this paper can be accessed here:

http://www.biomedcentral.com/1471-5945/13/14/prepub

## Supplementary Material

Additional file 1 Questions from the questionnaire 2008. Click here for file
