# Supplementary material for: Prevalence, incidence and predictive factors for hand eczema in young adults – a follow-up study
Source: BMC Dermatol. 2013 Oct 29;13:14. doi: 10.1186/1471-5945-13-14 (PMC3819704; doi:10.1186/1471-5945-13-14)
Supplement: Additional file 1 — Questions from the questionnaire 2008. [file 1471-5945-13-14-S1.doc]

**Appendix: Questions from the questionnaire 2008.**

**Year of birth: 19___** Male  Female

Number of persons in the household, yourself included**? _____** Number of children 0 - 4 years of age**?** _____

**How many hours a day do you usually spend:**
- cook _____ *(hours)*
- cleaning/washing laundry _____ *(hours)*
- taking care of children 0 – 4 years _____ *(hours)*

**How many hours a week on average have you done the following in the last 12 month?**- gardening (during summer season):_____ *(hours)*- repairing cars/engines: _____*( hours)*- building work, restoration: _____*( hours)*- sports/athletics: ____*( hours*) **What kind**?______________________________________________- hobbies: ____*( hours*) **What kind**?__________________________________________________

**What is your profession? _____________________________________ Since when? _____** *(year)*

**What are your main working tasks? ______________________________________________________________Since when? ____** *(year)*

Number of working hours at ordinary work**?** _____*(hours/week)*

Number of working hours at additional work**?** _____ (*hours/week*)

Number of times a day washing hands**?** **At home**:_____Times/day **At work**:_____ Times/day **Did you have eczema in your childhood?** yes  no  I do not know **Have you ever had asthma?** yes  no  I do not know  **Have you ever had allergic symptoms in your nose or eyes?** yes  no  I do not know  **How often do you use moisturisers?** daily  some time each week  some time each month  never

**How many hours a day do you use protective gloves?** _____ (hours)

**Have you ever had hand eczema?** yes  no  I do not know

**When did you have hand eczema?**- I have it at the moment  - not now, but within the last three month  - 3-12 month ago  - more than 12 month ago
